# Low transmission of SARS-CoV-2 derived from children in family clusters: An observational study of family households in the Barcelona Metropolitan Area, Spain

**DOI:** 10.1371/journal.pone.0277754

**Published:** 2022-11-17

**Authors:** Maria Mele-Casas, Cristian Launes, Mariona F. de Sevilla, Maria Hernandez-Garcia, Gemma Pons-Tomas, Quique Bassat, Victoria Fumado, Claudia Fortuny, Aleix Garcia-Miquel, Elisenda Bonet-Carne, Clara Prats, Sara Ajanovic, Marta Cubells, Joana Claverol, Daniel Penela-Sanchez, Cristina Jou, Sara Arias, Nuria Balanza, Barbara Baro, Pere Millat-Martinez, Sergio Alonso, Enric Alvarez-Lacalle, Marti Catala, Daniel Cuadras, Carmen Muñoz-Almagro, Eduard Gratacos, Iolanda Jordan, Juan Jose Garcia-Garcia

**Affiliations:** 1 Pediatric Department, Hospital Sant Joan de Déu, University of Barcelona, Barcelona, Spain; 2 Institut de Recerca Sant Joan de Déu, University of Barcelona, Barcelona, Spain; 3 Consorcio de Investigación Biomédica en Red de Epidemiología y Salud Pública (CIBERESP), Madrid, Spain; 4 ISGlobal, Hospital Clínic—Universitat de Barcelona, Barcelona, Spain; 5 Centro de Investigação em Saúde de Manhiça (CISM), Maputo, Mozambique; 6 ICREA, Pg. Lluís Companys 23, Barcelona, Spain; 7 Infectious Diseases Department, Hospital Sant Joan de Déu, Barcelona, Spain; 8 BCNatal | Fetal Medicine Research Center (Hospital Clínic and Hospital Sant Joan de Déu), University of Barcelona, Barcelona, Spain; 9 Institut d’Investigacions Biomèdiques August Pi i Sunyer (IDIBAPS), Barcelona, Spain; 10 Universitat Politècnica de Catalunya, BarcelonaTech, Barcelona, Spain; 11 Department of Physics, Computational Biology and Complex Systems (BIOCOM-SC), Universitat Politècnica de Catalunya, Castelldefels, Spain; 12 Fundació Sant Joan de Déu, Barcelona, Spain; 13 Pediatric Intensive Care Unit, Hospital Sant Joan de Déu, University of Barcelona, Barcelona, Spain; 14 Department of Pathology and Biobank, Hospital Sant Joan de Déu, Barcelona, Spain; 15 CIBERER, Instituto de Salud Carlos III, Barcelona, Spain; 16 Centre for Comparative Medicine and Bioimage (CMCiB), Germans Trias i Pujol Research Institute (IGTP), Badalona, Spain; 17 Statistics Department, Fundació Sant Joan de Déu, Barcelona, Spain; 18 Department of Medicine, Universitat Internacional de Catalunya, Barcelona, Spain; Duta Wacana Christian University School of Medicine / Bethesda Hospital, INDONESIA

## Abstract

**Background:**

Family clusters offer a good opportunity to study viral transmission in a stable setting. We aimed to analyze the specific role of children in transmission of SARS-CoV-2 within households.

**Methods:**

A prospective, longitudinal, observational study, including children with documented acute SARS-CoV-2 infection attending 22 summer-schools in Barcelona, Spain, was performed. Moreover, other patients and families coming from other school-like environments that voluntarily accessed the study were also studied. A longitudinal follow-up (5 weeks) of the family clusters was conducted to determine whether the children considered to be primary cases were able to transmit the virus to other family members. The household reproduction number (Re*) and the secondary attack rate (SAR) were calculated.

**Results:**

1905 children from the summer schools were screened for SARS-CoV-2 infection and 22 (1.15%) tested positive. Moreover, 32 additional children accessed the study voluntarily. Of these, 37 children and their 26 households were studied completely. In half of the cases (13/26), the primary case was considered to be a child and secondary transmission to other members of the household was observed in 3/13, with a SAR of 14.2% and a Re* of 0.46. Conversely, the SAR of adult primary cases was 72.2% including the kids that gave rise to the contact tracing study, and 61.5% without them, and the estimated Re* was 2.6. In 4/13 of the paediatric primary cases (30.0%), nasopharyngeal PCR was persistently positive > 1 week after diagnosis, and 3/4 of these children infected another family member (p<0.01).

**Conclusions:**

Children may not be the main drivers of the infection in household transmission clusters in the study population. A prolonged positive PCR could be associated with higher transmissibility.

## Background

As of October 2022, SARS-CoV-2 has infected >600 million people resulting in >6.5 million deaths according to the World Health Organization (WHO).

At the beginning of the pandemic, in a scenario of uncontrolled transmission, stringent lockdown measures were adopted globally. Such measures were particularly strict for children, including the early closure of schools, with rigorous confinement and the recommendation to avoid contact with the elderly [1, 2]. The adoption of such measures was based on the existing knowledge about children as the main drivers of other respiratory viral infections, such as influenza virus [3]. According to this assumption, children could act as the main spreaders of SARS-CoV-2, and until proven to the contrary, they needed to be targeted as a high priority for confinement [4].

Nonetheless, current understanding of SARS-CoV-2 transmission suggests that the contribution of children to the overall community transmission may be minor [5–7]. An epidemiological study at the beginning of the pandemic in Spain showed that children and adolescents had lower seroprevalence than adults (3.4% vs 6.0% in adults older than 65) [8]. However, recent epidemiological data from the CDC show that the number and rate of cases in children in the United States have been steadily increasing since March 2020, from 46 incident cases/100,000 population to 169 incident cases/100,000 population in November 2021 [9]. Therefore, transmissibility and the role of children in SARS-CoV-2 infection spreading in the community still remain controversial [10].

For this reason, this study has focused on children, a population less studied and highly affected during the pandemic, aiming to identify differences in their transmission potential in relation to adults. Thus, the primary aim of this study was to determine the role of children in the transmission of SARS-CoV-2 in households. Household transmission clusters allow the evaluation of viral transmission and susceptibility to infection [8, 11], as the household is a stable setting, with close contact between cohabitants and without restrictive measures.

## Methods

### Study description

This was a prospective, longitudinal, observational study, including children attending 22 summer schools in the Barcelona Metropolitan Area, Spain. Children from other similar school-like environments (see inclusion and exclusion criteria) were included in the study voluntarily. The recruitment period ran from 29^th^ June to 31^st^ July 2020. Parents and siblings from children with confirmed SARS-CoV-2 infection were screened and evaluated as close contacts and followed up for 5 weeks. Four steps in the study of each household were followed: 1) identification of infected children from the study setting (index case), 2) study of the household-based family contacts of these positive children, 3) clinical and epidemiological data collection through a structured questionnaire, and finally 4) longitudinal follow up of those family contacts to determine whether the children who were primary cases were able to transmit the virus to other family members.

### Inclusion and exclusion criteria

#### Inclusion criteria

Children aged 3 to 15 with detection of SARS-CoV-2 RNA in nasopharyngeal swab or saliva specimens using a real-time PCR (RT-PCR) (index cases) and their household contacts, regardless of age. These children came via three different recruitment pathways (RP): RP1/ Active surveillance cohort study in 22 summer schools, consisting of a longitudinal follow-up of a large number of children with weekly screening for SARS-CoV-2 infection (RT-PCR in nasopharynx or saliva) [12], RP2/ Cases identified by the Catalonian Health Surveillance System of SARS-CoV-2 infection diagnosed by nasopharyngeal RT-PCR while attending other summer schools or children’s foster homes in the area of Barcelona. These included children that were tested for SARS-CoV-2 PCR based on symptoms or because of a positive contact case; and RP3/ Individual cases referred from a public call made to enroll children with positive RT-PCR in the preceding 5 days. The 22 summer schools enrolled in the study (RP1) were located in 27 different locations within Catalonia, and were considered to be a representative sample of the region under study. RP2 and RP3 were included at the beginning of the study design due to the uncertainty in the evolution of the pandemic at that moment. If there had been a very low incidence, there would have been cases from that call based on passive surveillance.

#### Exclusion criteria

Children with infection at an earlier or previous unknown time, defined by the presence of positive Anti‐SARS‐CoV‐2 IgG. Households with incomplete follow up or from whom informed consent was not obtained.

### Definitions

#### Primary household cases

Children were considered primary cases independently of having or not having clinical symptoms if SARS-CoV-2 RT-PCR was detected in them in the absence of Anti‐SARS‐CoV‐2 IgG. Their household contacts needed to fulfill the following criteria at the time of the child diagnosis: absence of clinical symptoms, negativity for SARS-CoV-2 in RT-PCR testing and for Anti‐SARS‐CoV‐2 IgG and IgM.

Adults were considered primary cases if they were positive for SARS-CoV-2 and had the onset of symptoms before the index case (defined as more than two days), or if they had positive Anti‐SARS‐CoV‐2 IgG at the time of inclusion in the study, and the index case was negative for Anti‐SARS‐CoV‐2 IgG detection.

An indeterminate primary case was considered whenever the index case and their contacts were asymptomatic and when SARS-CoV-2 was detected at the time of inclusion to the study in more than one member of the family.

#### Secondary household cases

A new secondary incident infection was defined by one of the following conditions: 1) Negative RT-PCR and Anti‐SARS‐CoV‐2 IgG tests at enrolment followed by a positive test at 7 or 14 days, 2) Evidence of seroconversion at week 5 in the presence of a negative Anti‐SARS‐CoV‐2 IgG and with a negative baseline RT-PCR.

### Outcomes

The primary outcome of the study was the rate of transmission derived from pediatric primary cases to other children and adults, in the household setting (household reproduction number, Re* [13]). We calculated the Re*_children_ (total number of secondary cases inside household/total number of pediatric primary cases) and the Re*_adults_ (total number of secondary cases inside household/total number of adult primary cases).

We also calculated the secondary attack rate (SAR) that was defined as the ratio:

- SAR in children primary cases: (number of secondary infections)/(number of contacts evaluated) x100.
- SAR in adult primary cases: (number of secondary infections)/(number of contacts evaluated) x100.
- SAR in adult primary cases without including the index cases: (number of secondary infections—index cases)/(number of contacts evaluated) x100.

### Samples and laboratory measurements

Laboratory tests performed on participants are shown in Table 1.

**Table 1:** Sequence of laboratory specimen collection, tests, and follow-up visits after the diagnosis of the index case.

	Week 1	Week 2	Week 3 (14^th^ day)	Week 4	Week 5
**Index case**	Nasopharyngeal PCR Quick serology Serum serology	Nasopharyngeal PCR	Nasopharyngeal PCR		Nasopharyngeal PCR (only if 14^th^ day nasopharyngeal PCR was positive) Serum serology
**Household child contacts**	Nasopharyngeal PCR Quick serology	Nasopharyngeal PCR	Nasopharyngeal PCR	Nasopharyngeal PCR (only if 14^th^ day nasopharyngeal PCR was positive)	Nasopharyngeal PCR (only if previous nasopharyngeal PCR was positive) Quick serology
**Household adult contacts**	Nasopharyngeal PCR Quick serology Serum serology	Nasopharyngeal PCR	Nasopharyngeal PCR	Nasopharyngeal PCR (only if 14^th^ day nasopharyngeal PCR was positive)	Nasopharyngeal PCR Quick serology Serum serology

Nasopharyngeal swabs or nasopharyngeal aspirates were collected by testing teams, each composed of 2 research nurses. The swab was inserted along the nasal septum to the nasopharynx, until resistance was felt. It reached a depth equal to the distance from the nostrils to the earlobe. It was left in place for several seconds and then it was removed while rotating. For the nasopharyngeal aspirate, a disposable catheter connected to a vacuum source was inserted into one nostril until reaching the nasopharynx. The distance from the earlobe to the tip of the patient’s nose was the length to which the catheter was inserted. Secretions were recovered into a sterile container applying suction while the catheter was drawn back. The procedure was repeated with the same catheter and container in the other nostril. Samples were transported in viral inactivation transport medium. SARS-CoV-2 RNA detection was performed in these samples using different commercial *in vitro* diagnostic tests (TaqPath COVID-19 CE-IVD RT-PCR Kit, Thermofisher; Genefinder™ Plus RealAmp Kit, Genefinder laboratories and Allplex™ 2019-nCoV Assay—multiplex Real-time PCR assay, Seegene Laboratories) and were processed in the Orfeo Program according to CDC-006-00019 CDC/DDID/NCIRD/ Division of Viral Diseases protocol, available at https://www.fda.gov/media/134922/download.

Rapid IgG/IgM COVID-19 tests (Sure Screen) were performed according to manufacturer’s instructions in finger-prick capillary blood specimens. Serum samples were tested with an enzyme linked immunoassay (ELISA) (Euroimmune). Both quick and serum serology were performed by nursing staff.

### Statistical analysis

Chi-square test was used for comparisons of categorical data, and Student’s t-test or Mann-Whitney U for quantitative variables, according to their normal distribution or not. The Wilcoxon signed range test was used to compare paired numerical data. SPSS® 22.0 statistical package (IBM Corp. software, Armonk, NY) was used.

### Ethics

The study was approved by the Institutional Review Board and the Sant Joan de Déu Hospital Ethics Committee (PIC-140-20). All participants or their legal guardians provided written informed consent.

## Results

**Fig 1:**
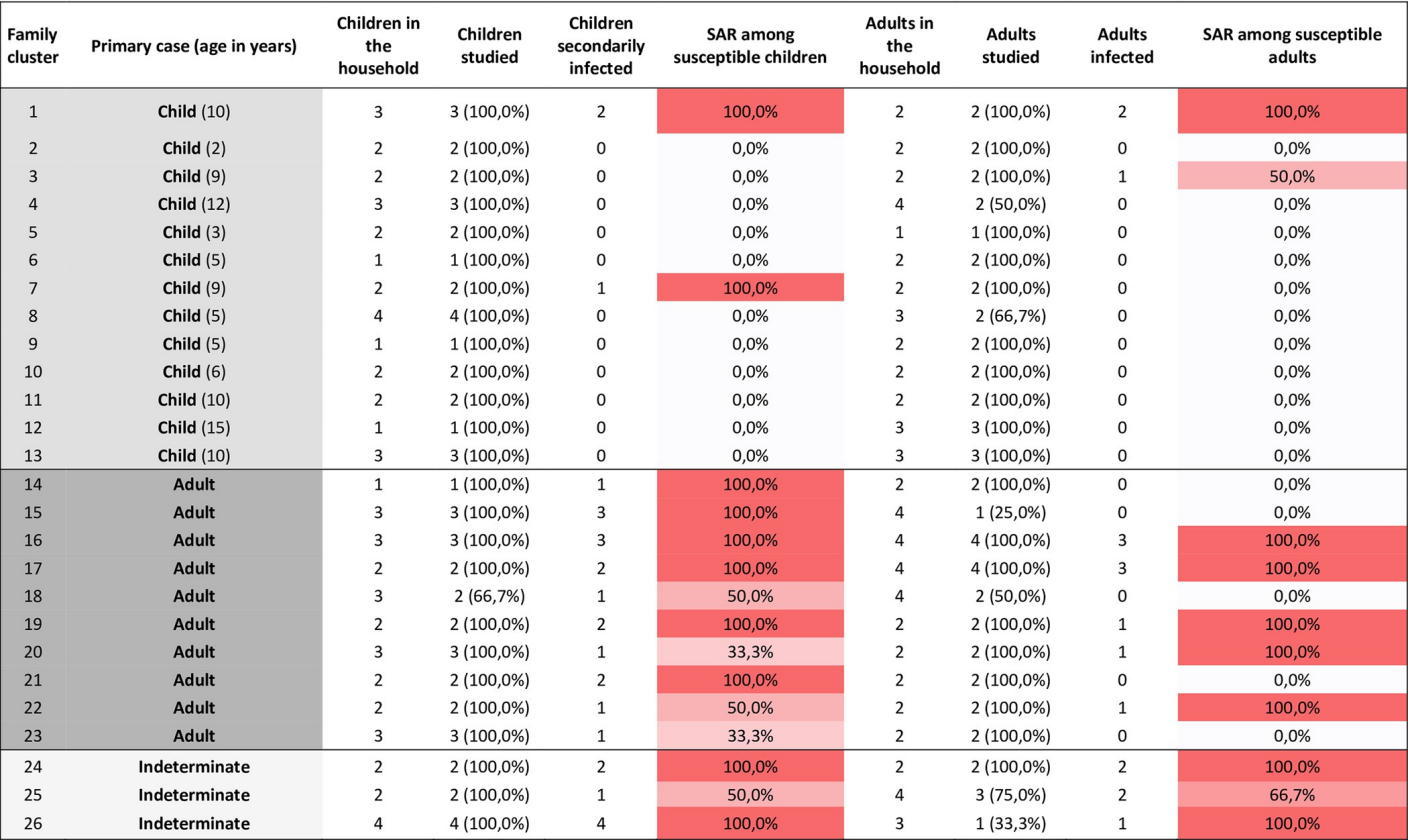
Number of inhabitants and SAR in each household according to the age-range of the primary case.

From the summer school cohort (RP1), 1905 children were studied, and SARS-CoV-2 was detected by RT-PCR in 22 (1.1%). Thirty-two other children were also diagnosed with SARS-CoV-2 infection (RP2 12, and RP3 20). Of those 54 index cases, 37 (68.5%) agreed to be studied, corresponding to 26 different households (Fig 1). Ninety household contacts were identified, of whom 78 (86.7%) agreed to be studied (22/23, 95.6% of children and 56/67, 83.6% of adults). In 20 households, 100.0% of the cohabitants were studied.

The median age of the index cases was 9 years old (IQR 5–11), and 54.0% were males (20/37). 64.9% presented symptoms (24/37), and none of them required hospitalization. The median number of household members was 4 (IQR 4–6): 2 children (IQR 2–3) and 2 adults (IQR 2–3).

**Fig 2:**
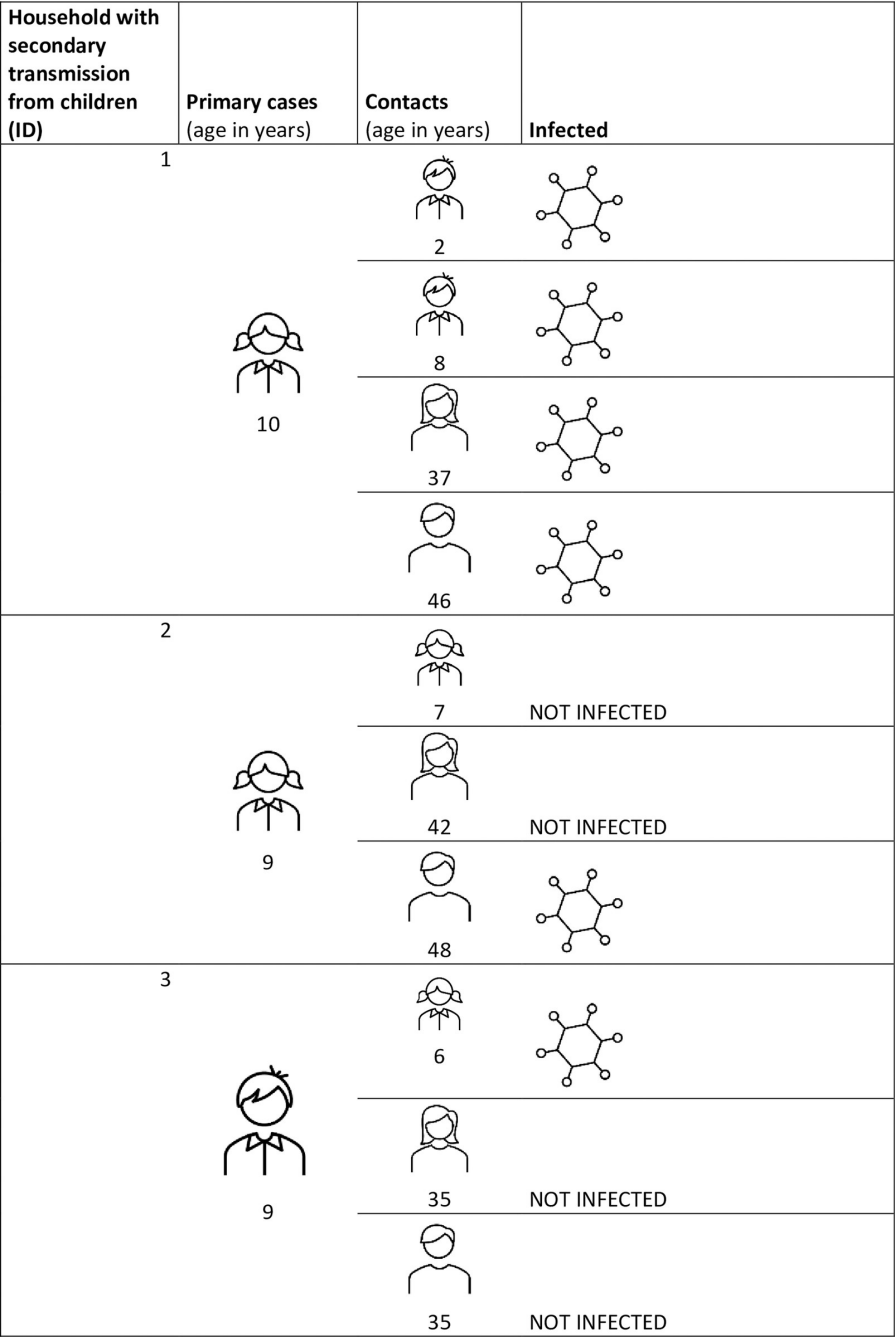
Description of the family clusters in which children infected other members of the household.

In 13 of the 26 studied households (50.0%), the primary case was a child, and transmission to other members was observed in 3/13 households (23.0%) (Fig 2). In one case, all the cohabitants (2 adults and 2 children) were presumably infected after the primary pediatric case. In another case, the child only infected one of the two parents. Finally, the last child infected his sister, but none of the adults.

Of note, in 9/13 of the pediatric primary cases (69.0%) nasopharyngeal PCR became negative only one week after the diagnosis. In those who had persistent SARS-CoV-2 RNA detection one week after diagnosis, the rate of children who infected another household contact was significantly higher (3/4 vs 0/9, p < 0.01). No other statistically significant differences were found between pediatric primary cases with demonstrated secondary transmission and those where transmission was not documented (Table 2).

**Table 2:** Comparison of demographic, clinical, and analytical variables of paediatric patients who transmitted the infection to other household members and those who did not transmit it.

	Total (n = 13)	Paediatric primary household case with demonstrated secondary transmission (n = 3)	Paediatric primary household case with no demonstrated secondary transmission (n = 10)	p
**Age** (years) Median (IQR)	9 (IQR 5–11)	9 (IQR 9–9.5)	5.5 (IQR 4.5–10.5)	0.49
**Sex** (males) (n)	5	1	4	0.68
**Symptoms** (n)	11	3	8	0.57
• Fever	8	2	6	0.68
• Cough	2	1	1	0.42
• Sore throat	4	1	3	0.71
• Gastrointestinal	4	2	2	0.20
• Headache	2	0	2	0.57
• Exanthema	2	0	2	0.57
**Handwashing**				
• 3–5 times/day	9	2	7	0.70
• >5 times/day	4	1	3	0.70
**Family members** median number (n)	4 (IQR 3.5–5.5)	4 (IQR 4–4.5)	4 (IQR 3–6.5)	0.81
**Household surface** (m^2^)	82 (IQR 61–120)	130 (IQR 60–130)	80 (IQR 62–115)	0.27
**m** ^**2**^ **/cohabitants**	18.7 (IQR 15–31)	32 (IQR 23–32)	17 (IQR 15–27)	0.35
**Room sharing** (n)	6	1	5	1
**Number of toilets** (n)	1.5 (IQR 1–2)	2 (IQR 1.5–2.5)	1 (IQR 1–2)	0.42
**Persistent positive nasopharyngeal PCR a week after diagnosis** (n)	4	3	1	**<0.01**

In 10 of the 26 studied households (38.4%), the primary case was an adult who infected 16 other household members in addition to the index case. In 3 families the primary case could not be determined.

According to the results, the secondary attack rate of pediatric primary cases was 14.2%. In addition, the effective reproduction number when the primary case was the child was Re*_children_ = 0.46 (of 13 child primary cases, there were only 6 secondary cases).

The secondary attack rate of adult primary cases was 61.5% (72.2% including the index cases). The effective reproduction number was Re*_adults_ = 2.6 in those households where the primary case was an adult.

**Fig 3:**
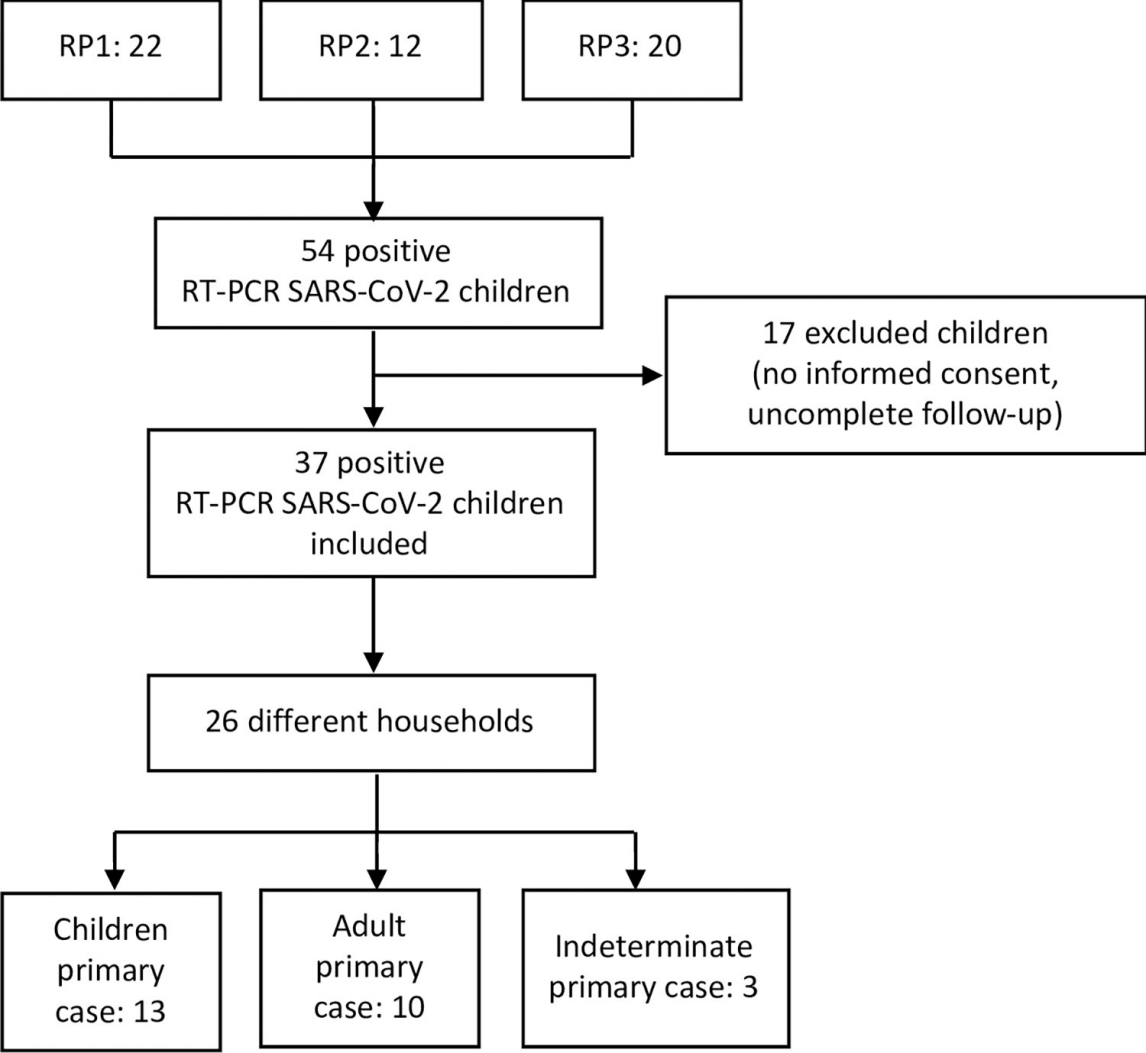
Flowchart including the different recruitment pathways, children included and excluded, and results after the contact tracing in the different family clusters.

The different recruitment pathways, children included and excluded, and results after the contact tracing in the different family clusters are illustrated in Fig 3.

## Discussion

There is still much uncertainty about the potential of children to act as effective transmitters of SARS-CoV-2 infections, with several studies showing lower transmission rates in comparison to adults and recent studies showing similar transmission rates [14–16].

The present study shows that the secondary attack rate (SAR) is much lower in children than in adults.

Within household clusters, it has been shown that children are at a higher risk of infection in comparison to other settings, but they are unlikely to be the primary drivers of the infection in the cluster [14, 15]. A recent meta-analysis found that in only 3.8% of the cases was a child determined to be the index case of the household cluster [17]. Indeed, this is consistent with our observation that while in 13 of the household clusters studied the primary case was a child, only 3 (23.0%) of them were shown to have fostered transmission to other members of the household.

In the same line, the secondary attack rate of child primary cases in this study was 14,2%. This value is in the lower range of the typical SAR in households, which has been estimated at 17,1% by a meta-analysis of 54 studies [18]. Furthermore, the effective reproduction number in the household when the primary case was the child was 0.46. This value is lower than the Re* measured in a household transmission study in the United States, which was around 0.9 [9]. More recently, other studies in the United States have shown similar rates of secondary infection among children and adults [16]. In our opinion, considering that all these studies were also conducted during the pre-variants period, the disparity in the results could be explained by differing setting variables, such as lifestyles and the main features of the houses (household surface, number of cohabitants per m^2^, number of toilets and rooms…), rate of household overcrowding, and also different COVID-19 restriction policies at the time of the study. Despite this, we did not find any difference in the main features of the houses and the number of individuals per home surface between families in which another member became secondarily infected and those in which there was no secondary infection. Recently, the emergence of the omicron variant has led to higher SARs both in children and adults. However, there is still no evidence that omicron preferably targets children more than other age groups [19]. However, vaccination rates were higher in older people since children got access to the vaccines much later, and this makes it very difficult to draw conclusions with regard to the role of children in the transmissibility of this variant.

A recent study noted that the secondary attack rate was lower in households with pediatric primary cases than those with adult primary cases (59% vs. 67.6%) [20]. In our study, the SAR of adult primary cases (72.2% including the kids that gave rise to the contact tracing study, and 61.5% without them) is completely biased since, by necessity, one pediatric case had already been detected. Therefore, an adult or someone else must have at least infected a child.

The fact that in the study population there were not households with zero infected kids may have led to an overestimation of the infectivity of adults as primary cases, resulting in a bias in estimating the secondary attack rates and having an impact on the main findings.

Children are susceptible to SARS-CoV-2 infection but they are more frequently asymptomatic or have mild symptoms compared to adults [21, 22]; this may partly explain why children are less likely to transmit the infection [17]. In our study, only 2 out of 13 primary cases in the household clusters were asymptomatic, but neither of these transmitted the infection to other household members.

Knowing the infectivity of people affected by the virus is especially important for the control of the pandemic, and one of the ways to investigate it relies on analyzing the viral load in the respiratory tract by RT-PCR. Lower risk of transmission may be due to lower viral loads but it is not certain that children have comparatively lower viral load levels than adults [23–25]. Related to this, we were not able to carry out the study of viral loads (due to the use of different commercial tests) but we did record when the negativization of the PCR of the study subjects took place. In 9 out of 13 primary cases, the PCR was negative one week after the diagnosis. Of the 4 remaining cases with a PCR persistently positive more than one week after the diagnosis, 3 of them infected a family member. Therefore, according to our findings, patients with prolonged positivity by PCR were associated with a higher secondary attack rate. According to some studies in adults, there seems to be a relationship between the severity of the disease and the longer detection of positive PCR [26]. Nevertheless, in the studies carried out so far, it could not be demonstrated that a persistently positive PCR was associated with increased infectivity [27]. To the best of our knowledge, there are no published studies discussing the relationship between persistence of a positive PCR and increased infectivity in children. This observation could be of interest in the future because the use of antivirals has been associated with faster viral clearance in adults [28]. Studies with new oral antivirals are needed to establish whether the use of these therapies is safe and effective for this purpose.

The present study has several limitations, such as the limited number of family clusters studied and the low number of cases of children considered to have been primary cases in the household. The fact that the main inclusion criteria was being a child with confirmed SARS-CoV-2 infection may have led to overestimation of the number of cases in which children were determined to be primary cases (13 out of 26), yet it might also have led to overestimation of the infectivity of the adults as primary cases (10 out of 26 primary cases were adults, and all of them transmitted the infection at least to one child). When in the same family unit there was more than one person infected, we were unable to rule out the transmission being from the primary case and not from one of the secondary cases. We were also unable to determine whether the infection of the household contacts was due to direct transmission inside the family unit, because we could not dismiss the community exposure as a putative source of infection. The children included in the study had a wide range of ages (3–15 years old) across different stages of education (kindergarten, primary school, and middle school), and were at different developmental stages having different behaviors and contact types, which might further differing risks of infections. However, we aimed to compare viral transmissibility between children and adults at the household level, as this is a stable setting, and the children index cases had homogeneous ages (70% between 5 and 10 years old). Moreover, the study took place at a time when masks were required outside the household and there were social restrictions. Another limitation was that we were unable to define the unique primary case when two members of the same cluster started symptoms within 1–2 days of each other (these cases have in fact been reported as indeterminate primary cases). Finally, we decided to use quick serologies in household child contacts to avoid venopuncture; the lower sensitivity of this method may have missed some infections. This potential misclassification of the outcomes may have affected the relative roles of children and adults in driving household transmission.

## Conclusions

Household clusters play an important role in the transmission of SARS-CoV-2. The present study confirms that the secondary attack rate (SAR) was much lower in children than in adults, and shows that the persistence of positive RT-PCR after one week appears to be associated with a higher rate of transmission at home. In our setting, children were not the main drivers of infection at the household level.
